# Efficacy of recombinant thrombomodulin for DIC after deceased donor liver transplantation: a case report

**DOI:** 10.1186/s40792-016-0208-8

**Published:** 2016-08-06

**Authors:** Koichi Kimura, Tomoharu Yoshizumi, Shinji Itoh, Norifumi Harimoto, Takashi Motomura, Noboru Harada, Akihisa Nagatsu, Toru Ikegami, Mizuki Ninomiya, Yuji Soejima, Yoshihiko Maehara

**Affiliations:** Department of Surgery and Science, Graduate School of Medical Sciences, Kyushu University, Fukuoka, 812-8582 Japan

**Keywords:** Deceased donor liver transplantation, Split graft, Recombinant thrombomodulin, Disseminated intravascular coagulation

## Abstract

**Background:**

Disseminated intravascular coagulation (DIC) after liver transplantation (LT) is a difficult complication. We report a case of disseminated intravascular coagulation after deceased donor liver transplantation (DDLT) treated with recombinant thrombomodulin (rTM).

**Case presentation:**

A 30-year-old woman underwent right tri-segment split graft DDLT for acute liver failure. She developed disseminated intravascular coagulation on post-operative day 5 with fever. Computed tomography revealed necrosis of hepatic segment IV, and her acute-phase disseminated intravascular coagulation score was seven points. She was given rTM, and the inflammation, liver function, and coagulation disorders immediately improved. However, pleural effusion drainage from the chest tube became bloody on post-operative day 11, and rTM was discontinued. She progressed well and was discharged from the hospital on post-operative day 28. rTM is an effective treatment for disseminated intravascular coagulation; however, rTM for cases with coagulation disorders, which can occur after liver transplantation, has both risks and benefits.

**Conclusions:**

We report a case of DIC after LT, in which rTM was potentially effective. Further studies are needed to determine the appropriate dosages, duration, and additional considerations for rTM therapy in liver transplantation patients.

## Background

Split liver transplantation (LT) is one of few surgical options to expand the donor pool and address organ shortages and increasing wait list mortality rates [1]. However, split LT is associated with problems related to the surgical technique and possible complications. Hong et al. reported that the adult 10-year patient survival rate was significantly lower for split extended right-liver graft compared with adult whole-liver and living donor right-liver graft [2]. Halac et al. showed that the most frequent complications in split LT were biliary complications, followed by vascular complications and segment IV necrosis [3]. These complications could lead to disseminated intravascular coagulation (DIC) and poor outcomes after split LT.

Recombinant thrombomodulin (rTM) has excellent anticoagulant activity and is a known therapy for DIC. Recent reports have shown the superiority of rTM over low-dose heparin for treating DIC and that rTM has several activities, including anti-inflammatory effects as well as its anticoagulant activity [4].

We present a patient with DIC who was treated with rTM after deceased donor liver transplantation (DDLT) with right tri-segment split graft.

## Case presentation

The patient was a 30-year-old female with jaundice. Blood analysis at a local hospital revealed severe liver failure, and she was transferred to our hospital. She was diagnosed as having acute liver failure of unknown etiology and underwent medical treatment, including plasma exchange and transfusion; however, her liver function did not improve. LT was planned, but there was no suitable living donor, and she was placed on the DDLT wait list. DDLT was performed with right tri-segment split graft 13 days later. At the time of abdominal closure, the transplanted graft showed ischemia in segment IV. The graft volume to the standard liver volume ratio was 123.4 % (graft volume = 1381 g), the cold ischemic time was 10 h and 32 min, the warm ischemic time was 50 min, and the anhepatic time was 1 h and 5 min. The native liver showed subtotal necrosis.

**Fig. 1:**
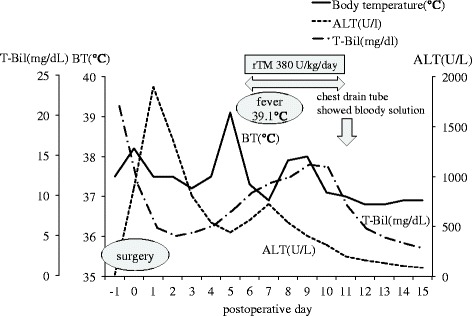
Post-operative changes in body temperature, total bilirubin levels, and alanine aminotransferase

**Fig. 2:**
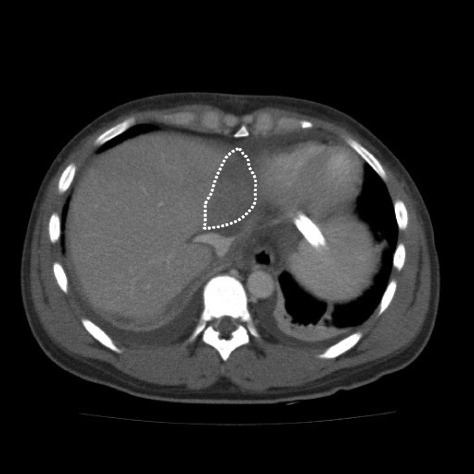
Computed tomographic images on POD 6 showing necrosis of segment IV of the transplanted graft

**Fig. 3:**
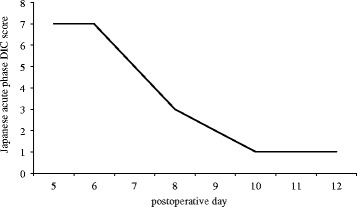
Japanese acute-phase DIC scores from on POD 5 to 12

Although no severe complications occurred after surgery, she developed a fever and increased total bilirubin level on post-operative day (POD) 5 (Fig. 1). Blood analysis revealed decreased platelets, coagulation abnormalities, and severe inflammation. Computed tomography on POD 6 revealed necrosis of segment IV of the transplanted graft (Fig. 2). She was diagnosed as having DIC based on the Japanese acute-phase DIC score (seven points) [5], and rTM was begun at 380 U/kg/day. Thereafter, inflammation, liver function, and coagulation abnormalities dramatically improved and the fever resolved (Figs. 1 and 3). However, on POD 12, the pleural effusion drainage from the chest tube became bloody. The anticoagulant effect of rTM was considered the cause and was discontinued. On POD 14, the pleural effusion drainage became serous, and on POD 19, the quantity of pleural effusion was minimal and the chest tube was removed. Thereafter, she made good progress and was discharged from hospital on POD 28.

### Discussion

rTM was effective for DIC after DDLT with right tri-segment split graft in this case, and we believe that necrosis of segment IV of the split graft caused the DIC.

DIC is a severe possible complication after LT and is a systemic disorder characterized by continuous intravascular coagulation related to an underlying disease. The liver produces most of the body’s coagulation factors, fibrinolytic compounds, and coagulation inhibitor factors. However, the dramatic coagulation disorders and fibrinolysis seen with DIC occur systemically after liver transplantation. Coagulation and fibrinolysis are markedly degraded in end-stage liver failure patients well before surgery, and these functions are degraded further after surgery because of the intra-operative anhepatic time and delayed time to improved graft function. After transplantation, the competing effects of acute inflammation with coagulation and fibrinolysis lead to thrombotic microangiopathy and systemic inflammatory response syndrome making it difficult to grasp the disease symptoms and treatment [6].

Cauley et al. [7] recently aimed to determine the current risk of graft failure in adult recipients after split LT by analyzing data from the United Network for Organ Sharing registry, assessing 889 split liver grafts performed from 1995 to 2010. Similar to previous analyses from the USA, the authors noted a significantly increased risk of graft failure in split grafts compared with whole grafts. In Japan, Sakamoto et al. showed that complications related to the operation were more frequent in split LT. They reported that the occurrence of bile leakage in extended right-lobe grafts was generally related to the viability of segment IV. The blood supply to segment IV, which arises primarily from the left-side vasculature, may be sacrificed during the splitting procedure and may increase the risk of parenchymal necrosis and bile leakage with an incidence of approximately 20–30 %. Segment IV-related complications may be directly associated with high rates of graft loss and mortality [8]. These data might include cases that develop DIC after surgery with necrotic areas in the graft, as in our case. Post-operative DIC can also occur following hepatectomy because liver partition and portal vein ligation for staged hepatectomy can cause post-operative ischemia in segment IV [9]. Monitoring hepatectomy patients for post-operative DIC is important with intra-operative visible hepatic ischemia.

rTM is a novel anticoagulant agent composed of the active, extracellular domain of thrombomodulin that regulates the imbalanced coagulation system by reducing excessive activation of thrombin [10]. In Japan, it has been reported that rhTM potentially reduces the morbidity and mortality in patients with sepsis-induced DIC [11–13]. Similarly, an international phase IIb clinical trial of rTM in patients with sepsis and suspected DIC [14] suggested that this treatment is both efficacious and safe, stimulating enthusiasm for the application of rTM in critical care. rTM also has an inhibitory effect on high-mobility group box 1 (HMGB1) by an anti- inflammatory action [15]. HMGB1 is an intranuclear protein that was originally identified as a DNA-binding protein but has since been recognized as a late-phase mediator during sepsis [16]. HMGB1 is also known to act as a pro-coagulant as well as a pro-inflammatory mediator for septic organ dysfunction. Hepatocyte nuclei contain high levels of HMGB1, which is released into the blood after ischemic-reperfusion injury to the liver and hepatocyte necrosis [17]. Therefore, rTM is a useful treatment for DIC after LT.

## Conclusions

In conclusion, we report a case of DIC after DDLT with right tri-segment split graft, in which rTM was potentially effective. Further investigations are needed to confirm the efficacy of rTM for DIC after LT and to consider rTM the standard treatment for DIC in LT recipients.

## Abbreviations

DDLT, deceased donor liver transplantation; DIC, disseminated intravascular coagulation; HMGB1, high-mobility group box 1; LT, liver transplantation; POD, post-operative day; rTM, recombinant thrombomodulin
